# Conventional Treatment for Multiple Myeloma Drives Premature Aging Phenotypes and Metabolic Dysfunction in T Cells

**DOI:** 10.3389/fimmu.2020.02153

**Published:** 2020-09-03

**Authors:** Rachel Elizabeth Cooke, Kylie Margaret Quinn, Hang Quach, Simon Harrison, Henry Miles Prince, Rachel Koldej, David Ritchie

**Affiliations:** ^1^Australian Cancer Research Foundation Translational Research Laboratory, Royal Melbourne Hospital, Melbourne, VIC, Australia; ^2^Department of Medicine, University of Melbourne, Melbourne, VIC, Australia; ^3^School of Health and Biomedical Sciences, RMIT University, Melbourne, VIC, Australia; ^4^Peter MacCallum Cancer Centre, Melbourne, VIC, Australia

**Keywords:** myeloma, T cell, metabolism, aging, autologous stem cell transplant

## Abstract

New diagnoses of multiple myeloma (MM) tend to occur after the age of 60, by which time thymic output is severely reduced. As a consequence, lymphocyte recovery after lymphopenia-inducing anti-MM therapies relies on homeostatic proliferation of peripheral T cells rather than replenishment by new thymic emigrants. To assess lymphocyte recovery and phenotype in patients with newly diagnosed MM (NDMM) and relapsed/refractory MM (RRMM), we tracked CD4^+^ and CD8^+^ T cell populations at serial time points throughout treatment and compared them to age-matched healthy donors (HD). Anti-MM therapies and autologous stem cell transplant (ASCT) caused a permanent reduction in the CD4:8 ratio, a decrease in naïve CD4^+^ T cells, and an increase in effector memory T cells and PD1-expressing CD4^+^ T cells. Transcriptional profiling highlighted that genes associated with fatty acid β-oxidation were upregulated in T cells in RRMM, suggesting increased reliance on mitochondrial respiration. High mitochondrial mass was seen in all T cell subsets in RRMM but with relatively suppressed reactive oxygen species and mitochondrial membrane potential, indicating mitochondrial dysfunction. These findings highlight that anti-MM and ASCT therapies perturb the composition of the T cell compartment and drive substantial metabolic remodeling, which may affect the fitness of T cells for immunotherapies. This is particularly pertinent to chimeric antigen receptor (CAR)-T therapy, which might be more efficacious if T cells were stored prior to ASCT rather than at relapse.

## Introduction

Multiple myeloma (MM) is a malignancy of plasma cells within the bone marrow (BM) (1, 2) that is typically diagnosed in patients over the age of 60 years old (3). Treatment for MM has evolved over the last 10 years with the introduction of immune modulatory drugs (IMiDs) and proteasome inhibitors. This has led to a significant increase in life expectancy (2, 4), which is most pronounced in patients under 60 years old (5). However, it is notable that the typical age of onset for MM coincides with marked decline in immune function during normal biological aging driven by thymic involution and immunosenescence.

From 60 years old, both the number and repertoire diversity of T_N_ cells declines substantially due to a process known as thymic involution (6, 7). Thymic involution refers to a loss of thymic stromal tissue and function, which limits *de novo* production of naïve T (T_N_) cells. With this decline in *de novo* T_N_ cell production, homeostatic proliferation of peripheral T cells appears to compensate and increases with age (8). As a result, in the event of a sudden decline in the number of lymphocytes (such as might occur during high dose chemotherapy), the aged thymus has limited capacity for T_N_ cell output (9, 10). Instead, repopulation of the peripheral T cell population is predominantly driven by lymphopenia-induced proliferation, mediated by the increased availability of γc cytokines, such as IL-7 and IL-15. Lymphopenia-induced proliferation favors expansion of CD8^+^ memory T cells, because CD8^+^ memory T cells express higher levels of a component of the IL-15 receptor (CD122) (11) and CD4^+^ T cell homeostatic expansion is limited by IL-7-dependent STAT-1 activation (12). More recently, signaling from γc cytokines has been seen to drive metabolic remodeling in T cells in mouse models of aging, inflammation, and lymphopenia (13–15), but the impact of lymphopenia-inducing therapies on T cell metabolism in aged humans has not been defined.

Immunosenescence refers to a loss of intrinsic function in immune cells, which can undermine responses to vaccines, infections, and cancer (16). Chronic age-related inflammation and metabolic stress are thought to be significant drivers of immunosenescence for a variety of immune cells, including CD4^+^ and CD8^+^ T cells (17, 18). During MM disease, it is well established that MM cells can create a microenvironment of chronic inflammation in the BM, characterized by increased production of IL-6 in particular (19). IL-6 sustains tumor survival, but it also drives production of senescent cells that exhibit a senescence-associated secretory phenotype (20, 21), all of which are predicted to augment dysfunction in CD4^+^ and CD8^+^ T cells. Inflammation-associated cytokine stimulation is also known to drive metabolic changes in a variety of immune cells, including T cells (13–15). Given the complex relationship between T cell homeostasis, inflammation, and aging, understanding the shifts in the immune system that result from normal aging, MM disease and MM therapies will be critical for implementing immune therapies in MM patients (22).

Previously, we demonstrated a loss of T_N_ cells in the peripheral blood (PB) of MM patients, with a reciprocal expansion of effector memory (T_*EM*_) cells (23). In the CD8^+^ T cell population, a loss of T_N_ cells is already observed at diagnosis and is equivalent to the loss of T_N_ cells seen in elderly healthy donors (HD). In contrast, in the CD4^+^ T cell population, T_N_ cells are relatively preserved in the elderly and newly diagnosed MM (NDMM), with significant loss of T_N_ cells only observed in relapsed/refractory MM (RRMM) (24). This also leads to a decrease in CD4:8 ratio, which is a characteristic finding in MM with disease progression (23, 25). While prior studies of patients with NDMM and RRMM concluded that changes in NK and T cell populations were corticosteroid-induced and/or disease-driven (23, 24, 26–28), autologous stem cell transplant (ASCT) and T cell-depleting therapies form the backbone of treatment for medically fit MM patients, and their long-term impact on T cell function has not been well-described.

In this study, we demonstrate that ASCT and/or MM disease does irreversibly change the composition of T cell populations, with a dramatic loss of CD4^+^ T_N_ cells and reciprocal increase in CD8^+^ effector memory T cells, and an increase in PD1-expressing CD4^+^ T cells. We also observe metabolic changes in T cells from RRMM patients, with increased expression of transcripts for mitochondrial respiration, increased mitochondrial load, and decreased reactive oxygen species (ROS), which suggests a loss of metabolic fitness. We propose a model where ASCT and/or MM disease-derived inflammation drives increased inflammatory and γc cytokine signaling and lymphopenia-induced proliferation and accelerated immunosenescence, which would mediate the dramatic loss of T_N_ cells in MM patients and metabolic remodeling in the remaining T cells. These findings inform our current treatment protocols for MM and will guide integration of new immune-based therapies for MM. While lymphopenia may drive CD4^+^ T_N_ cell loss, we may be able to intervene with dendritic cell (DC) vaccination, checkpoint blockade, or metabolic interventions during post-ASCT lymphopenia to better support the recovering T cell population. Furthermore, the collection of T cells prior to ASCT (as opposed to at the time of relapse) would likely provide a more efficacious chimeric antigen receptor (CAR)-T product.

## Materials and Methods

### Human Samples and Cells

**FIGURE 1:**
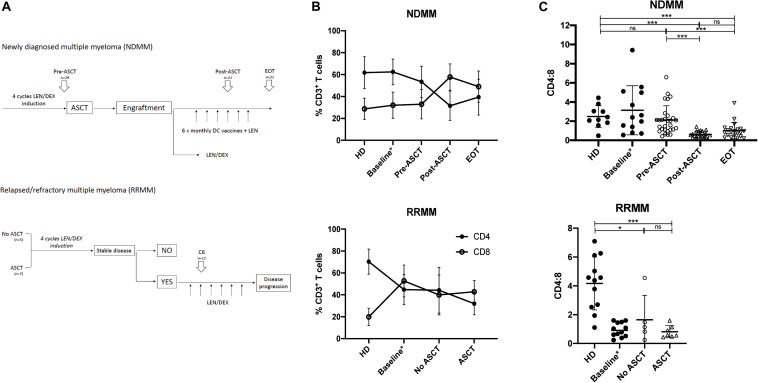
CD4:8 is reduced post-ASCT and in RRMM. **(A)** Time points analyzed by flow cytometry (described in section “Materials and Methods”). In newly diagnosed MM (NDMM), paired samples (where available) were analyzed prior to autologous stem cell transplant (pre-ASCT, *n* = 29), post-ASCT (*n* = 21) and at end of treatment (EOT, *n* = 21). In relapsed/refractory MM (RRMM), samples were analyzed after six cycles LEN/DEX and subdivided into those patients who had not had a prior ASCT (*n* = 5) and those with prior ASCT (*n* = 7). **(B)** The proportion of CD3^+^ T cells that are CD4^+^ (black circle) or CD8^+^ (clear circle) at serial time points in NDMM (top) and in RRMM (bottom) compared with HD. Baseline samples (NDMM *n* = 21, RRMM *n* = 10) analyzed in a prior study (23) are incorporated for interest. **(C)** CD4:8 ratio in NDMM (top) and in RRMM (bottom) compared with HD. Baseline samples (NDMM *n* = 21, RRMM *n* = 10) analyzed in a prior study (23) are incorporated for interest but are not included in statistical analysis. Statistical significance was determined using the Mann–Whitney test (*p < 0.05, ***p < 0.001).

All research was conducted in full compliance with principles of the “Declaration of Helsinki,” Good Clinical Practice, and within the laws and regulations of Australia. Cryopreserved peripheral blood mononuclear cells (PBMCs) were analyzed from MM patients enrolled on two historical clinical trials: LITVACC (ACTRN12613000344796) that recruited NDMM patients and REVLITE (NCT00482261) that recruited RRMM patients, which are illustrated in Figure 1A.

Newly diagnosed MM patients received four induction cycles of lenalidomide and dexamethasone (LEN/DEX) followed by ASCT, and they were then partitioned into one of two study arms, with either (i) monthly DC vaccines + LEN or (ii) LEN ± DEX maintenance therapy. Both study arms have been combined in this analysis as sample numbers were underpowered to detect any small variances. Paired patient PBMC samples (where available) were analyzed at three consecutive time points: cycle 4 of induction (Pre-ASCT, *n* = 29), at maintenance cycle 4 after ASCT (Post-ASCT, *n* = 21), and at last follow-up [end of treatment (EOT), *n* = 21]. The mean time to last follow-up was well over 2 years at 1069 days (range: 397–1780 days), to ensure maximal peripheral leukocyte reconstitution.

Relapsed/refractory MM patients were also treated with successive cycles of LEN/DEX and evaluated for stable disease, and for those who had stable disease, we analyzed PBMC samples after six cycles of LEN/DEX. We retrospectively subdivided this group for analysis into those patients who had not received prior ASCT (No ASCT, *n* = 5) and those who had (ASCT, *n* = 7).

Peripheral blood mononuclear cells from age-matched HD were used as a control group and were collected via the Australian Red Cross Blood Service and the Victorian Blood Donor Registry.

### T Cell Flow Cytometric Analysis

Flow cytometry was performed using Fixable viability stain 700 (BD Horizon) and directly conjugated anti-human antibodies: CD3 (SK7, BD Biosciences), CD4 (RPA-T4, BD Pharmingen or SK3, BD Horizon), CD8 (RPA-T8, BD Pharmingen), CD45RA (H100, eBiosciences), CD197 (CCR7) (3D12, eBiosciences) and CD279 (PD1) (EH12, BD Horizon). Cell surface staining was performed with 5 × 10^6^ to 1 × 10^7^ PBMCs in a 100-μl cell suspension of FACS buffer and antibody master mix for 30 min at 4°C. Cells were washed and resuspended in FACS buffer or complete RPMI containing mitochondrial fluorescent dyes for mitochondrial assessment. Lymphocytes were gated using forward and side scatter, followed by doublet and dead cell removal. BD Anti-Mouse or Anti-Goat Comp Beads were used to optimize fluorescence compensation settings for analysis. Fluorescence minus one (FMO) controls were used to assist with gating. Analysis was performed on a BD LSR Fortessa and analyzed using FlowJo software (Version 10). T cell differentiation subsets were defined as the following: Naïve (TN): CD3+CD4+/8^+^CD45RA^+^CCR7^+^, central memory (T_CM_): CD3^+^CD4^+^/8^+^CD45RA^–^CCR7^+^, effector memory (T_EM_): CD3^+^CD4^+^/8^+^CD45RA^–^CCR7^–^, and effector memory CD45RA^+^ (T_EMRA_): CD3^+^CD4^+^/8^+^CD45RA^+^CCR7^–^.

### RNA Sequencing

CD4^+^ T cells were enriched from PBMCs using the MACS^®^ human CD4^+^ T cell isolation kit, as per the manufacturer’s instructions. CD4^+^ T cell purity was >80%. RNA was extracted with Trizol, and cDNA library creation and RNA sequencing were performed by the Australian Genome Research Facility at the Walter and Eliza Hall Institute. Because there was significant RNA degradation in the sample set, the SMARTer Stranded Total RNA-Seq Kit—Pico Input Mammalian (Takara Bio USA) protocol was used, which incorporates a ribosomal RNA depletion. Genes needed to pass a 20 read threshold in >80% of samples in order to be analyzed. Bioinformatic analysis was performed at Melbourne Bioinformatics, University of Melbourne, utilizing the Limma linear model (29).

### Real-Time qPCR

CD4^+^ and CD8^+^ T cells were enriched from PBMCs using the MACS^®^ human CD4^+^ T cell isolation kit or MACS^®^ human CD8^+^ Microbeads (Miltenyi Biotec), as per the manufacturer’s instructions. RNA was extracted with Trizol and cDNA was synthesized using the Superscript VILO cDNA kit (Thermo Fisher Scientific), as per the manufacturer’s instructions. Target genes were analyzed using TaqMan gene expression assays with TaqMan Fast Universal master mix on the QuantStudio 7 Flex Real-Time PCR system (Thermo Fisher Scientific), as per the manufacturer’s instructions. The housekeeping gene TBP was used as the endogenous control to calculate relative gene expression.

### Mitochondrial Staining

After cell surface staining, cells were incubated at 37°C for 30 min in complete RPMI containing mitochondrial fluorescent dyes (Thermo Fisher Scientific), using a combination of MitoTracker deep red FM (MTdr) at 1:20,000 and either CellRox^®^ Orange reagent (CellRoxO) at 1:1000 or tetramethylrhodamine, methyl ester (TMRM) at 1:3000. Cell suspensions were washed and resuspended in T cell media for flow cytometric analysis.

### Statistical Analysis

Data were analyzed using GraphPad Prism software (version 7).

## Results

### Phenotypic Changes in the Composition of T Cell Subsets

To track changes in T cell populations throughout the course of MM disease and treatment, we analyzed PBMCs from patients with NDMM and RRMM collected at serial time points throughout their treatment and compared with age-matched HD. Study design and analysis time points are described in section “Materials and Methods” and in Figure 1A, and patient characteristics are shown in Supplementary Tables 1, 2. Unfortunately, baseline samples from patients prior to any treatment were not available for this analysis, but they have been described in previous work (23), which we include for interest but do not include in statistical analysis as these are not matched samples and it should be noted that different fluorochromes and gating strategies were used in the prior analysis.

In NDMM, the CD4:8 ratio was similar to HD in patients at baseline and pre-ASCT after four induction cycles of LEN/DEX, but it was significantly reduced post-ASCT and did not recover to baseline by EOT (Figures 1B,C, left). Prolonged CD4^+^ T cell depletion has been described after ASCT in children and young adults (10), but the CD4:8 ratio recovers to baseline levels by 1 year post-ASCT (30). Our results in an older population differ in this regard, and support findings by Chung et al. (30), who also demonstrated a reduction in the CD4:8 ratio in MM patients after ASCT up to 1 year post-ASCT. Subanalysis of the population that received DC vaccines (*n* = 8) suggested a trend toward expansion of the CD8^+^ T cell population (and further reduction of CD4:8) compared with those receiving LEN/DEX therapy (*n* = 13) at the post-ASCT time point; however, this did not reach statistical significance and was indistinguishable by EOT (Supplementary Figure 1).

In RRMM (at baseline and after LEN/DEX treatment, irrespective of prior ASCT), the CD4:8 ratio was also significantly reduced compared with HD (Figures 1B,C, right), although we note that the mean CD4:8 ratio in the this HD group is higher than that used for the NDMM comparison that may reflect heterogeneity in the normal population and/or minor differences in the fluorochromes used. The CD4:8 ratio is likely reduced in RRMM patients as a whole as they were probably treated previously with cyclophosphamide (commonly used in induction regimens in Australia), which is known to induce lymphopenia.

**FIGURE 2:**
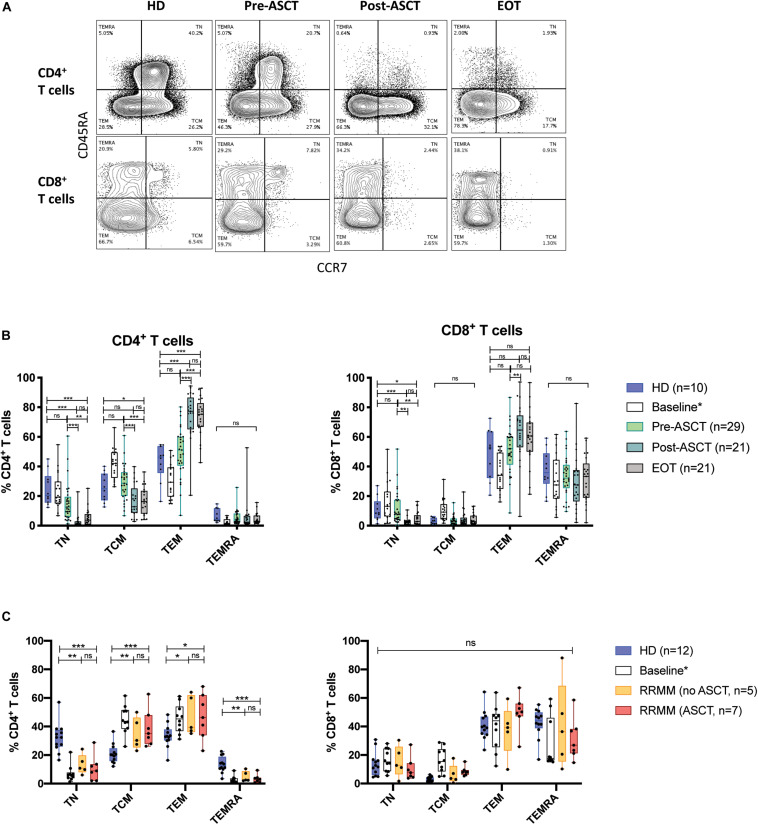
Phenotypic composition of T cell populations with MM. **(A)** Representative flow plots of CD4^+^ and CD8^+^ T cells from serial time points in NDMM samples, defining T_N_, T_CM_, T_EM_, and T_EMRA_ subsets. **(B,C)** The proportion of CD4^+^ (left) and CD8^+^ (right) T cells of each phenotype subsets at serial time points in **(B)** NDMM and **(C)** RRMM patient samples. Baseline samples analyzed in a prior study (23) are incorporated for interest but are not included in statistical analysis. Statistical significance was determined using multiple Student’s *t*-tests with the Holm–Sidak method applied (*p < 0.05, **p < 0.01, ***p < 0.001).

We next observed the differentiation status of CD4^+^ and CD8^+^ T cell populations over the course of treatment. In NDMM pre-ACST, phenotypic composition was similar to HD, but post-ASCT, there was a significant decrease in the proportion of CD4^+^ that were T_N_ and T_CM_ cells and a reciprocal increase in T_EM_ cells, which persisted to EOT (Figures 2A,B, left). In RRMM (at baseline and with treatment), there was also a significant decrease in the proportion of CD4^+^ T_N_ cells but, unlike in NDMM, both the CD4^+^ T_CM_ and T_EM_ populations had reciprocally expanded (Figure 2C, left). These shifts in phenotypic composition were not evident in CD8^+^ T cells, because T_EMRA_ cells are already an established subset in older HD (Figures 2B,C, right).

### There Are Temporal and Compartmental Differences in PD-1 Expression Between CD4^+^ and CD8^+^ T Cells

In the Vk^∗^MYC murine model of MM, PD1 expression was shown to transiently increase on T cells 30 days post-transplant (31). PD-L1 blockade also increased the efficacy of hematopoietic stem cell transplant, adoptive T cell transfer, and a post-transplant DC vaccine (31), and increased survival when administered during the homeostatic proliferation phase after non-myeloablative total body irradiation (32). This suggests that PD1/PD-L1 monoclonal antibodies may be beneficial post-ASCT, but human studies have not demonstrated significantly altered PD1 expression post-ASCT (30). We wanted to further dissect PD1 expression in CD4^+^ and CD8^+^ T cell subsets during MM treatment.

**FIGURE 3:**
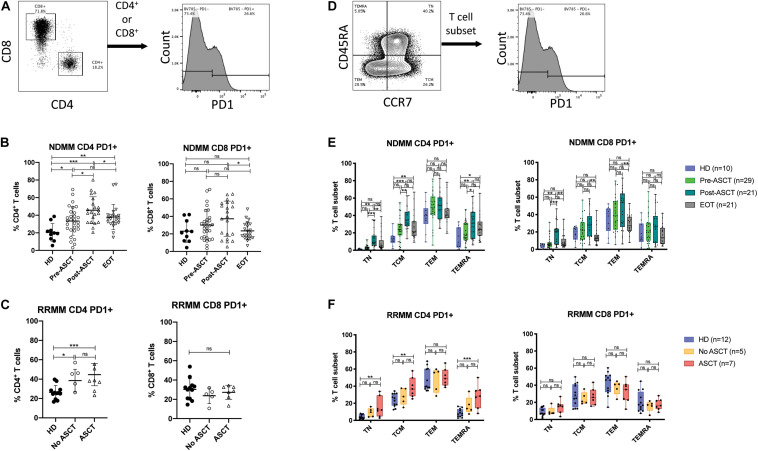
PD1 expression on T cell subsets after ASCT. **(A–C)** PD1 expression in CD4^+^ and CD8^+^ T cells. **(A)** Representative gating strategy. **(B,C)** Scatter plots showing PD1^+^ events as% of CD4^+^ T cells (left) and CD8^+^ T cells (right) in **(B)** NDMM at serial time points and **(C)** RRMM, compared to HD. Statistical significance was determined using the Mann–Whitney test (*p < 0.05, **p < 0.01, ***p < 0.001). **(D–F)** PD1 expression in T cell differentiation subsets. **(D)** Representative gating strategy. **(E,F)** Box and whisker plots showing PD1^+^ events as% of T cell subset (T_N_, T_CM_, T_EM_, and T_EMRA_) for CD4^+^ T cells (left) and CD8^+^ T cells (right) in **(E)** NDMM at serial time points and **(F)** RRMM, compared to HD. Statistical significance was determined using the Student’s *t*-test with the Holm–Sidak method applied (*p < 0.05, **p < 0.01, ***p < 0.001).

In CD4^+^ T cells, the proportion expressing PD1 was increased in both NDMM and RRMM patients compared with HD and was highest post-ASCT in NDMM (Figures 3B,C, left). In CD8^+^ T cells, there was no statistically significant change but there did appear to be two populations post-ASCT: one with and one without PD1 expression (Figures 3B,C, right). Subanalysis shows a trend toward increased % PD1^+^ in the group receiving DC vaccine but this does not reach statistical significance (Supplementary Figure 3). PD1 expression remained high in CD4^+^ T cells at EOT, while it returned to normal in CD8^+^ T cells (Figure 3B). The latter finding was also described in the post-allogeneic stem cell transplant setting, where PD1 facilitated apoptosis of alloreactive CD8^+^ T cells by increasing ROS (33).

PD1^+^ events were then backgated to define the phenotypic distribution of PD1 expression (Supplementary Figure 2). In HD, most PD1^+^ cells were T_EM_ phenotype, with minor proportions of T_N_, T_CM_, and T_EMRA_ phenotypes; however, there were significant proportions of T_CM_ and T_EMRA_ in CD4^+^ and CD8^+^ PD1^+^ populations, respectively. We then assessed PD1 expression in T cell differentiation subsets (Figure 3D). In CD4^+^ T cells, the proportion of T_N_, T_CM_, and T_EMRA_ that were PD1^+^ increased significantly in NDMM post-ASCT and in RRMM with prior ASCT, compared with HD (Figures 3E,F, left). The proportion of CD4^+^ T_CM_ that were PD1^+^ also remained elevated at EOT in NDMM. In CD8 T cell subsets, differences in PD1 expression were modest (Figures 3E,F, right), in keeping with prior observations (30).

### Fatty Acid β-Oxidation Is Increased in T Cells From RRMM Compared With HD

T cell function and survival are supported by the selective use of distinct metabolic pathways (34–36). For example, effector T cell activation and proliferation is supported by a shift toward aerobic glycolysis, while memory T cells utilize fatty acid oxidation (FAO) to fuel mitochondrial respiration. Furthermore, as previously mentioned, animal models demonstrate that aging, inflammation, and lymphopenia can all alter the metabolic capacity of T cells to promote mitochondrial respiration (13–15).

We performed preliminary RNA-Seq analysis on CD4^+^ T cells from HD (*n* = 2) and RRMM (*n* = 2) samples to identify differentially expressed genes (DEGs). When lists of DEGs were interrogated for significant enrichment of specific biological pathways in the KEGG databases, the top upregulated pathways included RAP1, PI3K-AKT, cAMP, calcium, and PPAR signaling pathways (Supplementary Figure 4), which can augment specific metabolic responses, particularly in relation to FAO and mitochondrial respiration.

**FIGURE 4:**
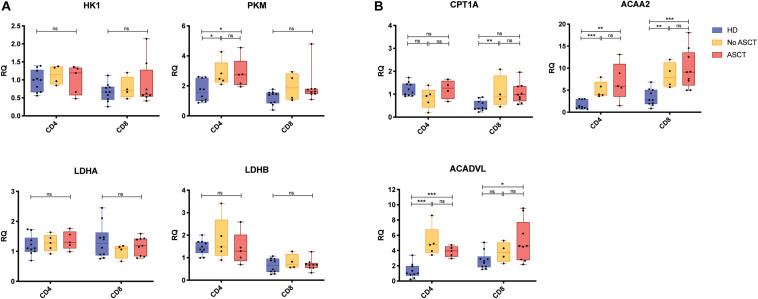
Expression of metabolic genes in CD4^+^ and CD8^+^ T cells. Relative transcript expression assessed by qPCR for **(A)** glycolysis-associated genes and **(B)** FAO-associated genes. Study groups: Healthy donor (HD) (*n* = 12), RRMM No ASCT (*n* = 5)^1^, RRMM ASCT (*n* = 9)^2^. Statistical significance was determined using the Mann–Whitney test (*p < 0.05, **p < 0.01, ***p < 0.001). ^1^Insufficient RNA extracted from one CD8^+^ sample for analysis. ^2^Insufficient RNA extracted from four CD4^+^ samples for analysis.

To further explore this, we evaluated the expression of key genes for glycolysis and FAO (Table 1) in CD4^+^ and CD8^+^ T cells with a larger cohort of RRMM and HD samples by using quantitative PCR; sample characteristics are described in Supplementary Table 3. There was no difference in relative RNA quantitation (RQ) between RRMM and HD for nearly all of the glycolysis genes analyzed; the one exception was *PKM* (pyruvate kinase), which was increased in RRMM compared with HD (Figure 4A). While pyruvate kinase is in the glycolytic pathway, it generates pyruvate that can either undergo aerobic glycolysis or be converted into acetyl CoA to provide substrate for mitochondrial respiration. In contrast, the expression of nearly all of the FAO genes were significantly higher in both CD4^+^ and CD8^+^ T cells in RRMM compared to HD (Figure 4B). These results suggest that FAO and mitochondrial respiration is upregulated in T cells in RRMM compared with HD.

**TABLE 1:** Genes analyzed by qPCR to assess glycolysis and fatty acid β-oxidation in T cells.

Mode of metabolism	Gene	Abbreviation	Function
Glycolysis	Hexokinase 1	HK1	Hexokinases phosphorylate glucose to produce glucose-6-phosphate, the first step of glycolysis. This gene encodes a ubiquitous form that localizes to the outer membrane of mitochondria.
	Pyruvate kinase, muscle	PKM	Enzyme involved in the production of pyruvate that can either undergo aerobic glycolysis or be converted to acetyl CoA (a substrate of the TCA cycle)
	Lactate dehydrogenase, A and B subunits	LDHA, LDHB	Catalyze the interconversion of pyruvate and lactate in the last step of aerobic glycolysis
Fatty acid β-oxidation	Carnitine palmitoyl transferase 1A	CPT1A	Rate-limiting enzyme in the transport of fatty acids across the mitochondrial inner membrane
	Acyl-CoA dehydrogenase, very long chain	ACADVL	Catalyzes the first step of the mitochondrial fatty acid β-oxidation pathway
	Acetyl-CoA acyltransferase 2	AACA2	Catalyzes the last step of the mitochondrial fatty acid β-oxidation pathway

### Mitochondrial Mass Is Significantly Increased in T Cell in RRMM

**FIGURE 5:**
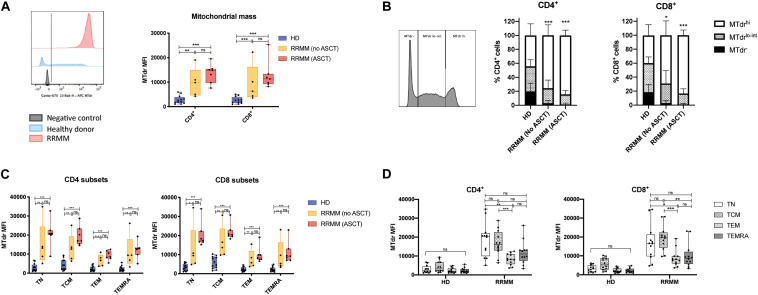
Mitochondrial mass in RRMM. **(A)** Mitochondrial mass measured by geometric mean fluorescence intensity (MFI) of MitoTracker Deep Red (MTdr) in CD4^+^ and CD8^+^ T cells from healthy donors and RRMM. Representative histograms (left) and box and whisker plots (right). **(B)** Proportion of CD4/8 T cells with high (MTdr^hi^), low-intermediate (Mtdr^lo–int^), and absent (MTdr^–^) mitochondrial mass by MFI. Representative histogram (left) and stacked bar charts (right). **(C,D)** Box and whisker plots showing mitochondrial mass by MFI in CD4^+^ and CD8^+^ T cell subsets from RRMM and HD by **(C)** treatment group or by **(D)** T cell subset (with no ASCT and ASCT combined for RRMM). Study groups as described in Figure 1. Statistical significance in all figures was determined using multiple Student’s *t* tests with the Holm–Sidak method applied (*p < 0.05, **p < 0.01, ***p < 0.001).

Given that genes associated with FAO and mitochondrial respiration were upregulated in T cells in RRMM, we then assessed mitochondrial mass using the MitoTracker^TM^ Deep Red FM dye (MTdr). MTdr staining was significantly increased in both CD4^+^ and CD8^+^ T cells from RRMM patients compared with HD (Figure 5A); in fact, virtually all T cells had high MTdr expression, unlike HD, where T cells were predominantly MTdr low-intermediate or MTdr negative (Figure 5B). This increase in MTdr staining was seen across all T cell subsets in RRMM (Figure 5C), but it was most marked in T_N_ and T_CM_ cells (Figure 5D).

### Mitochondrial ROS Levels Are Reduced in T Cells in RRMM

**FIGURE 6:**
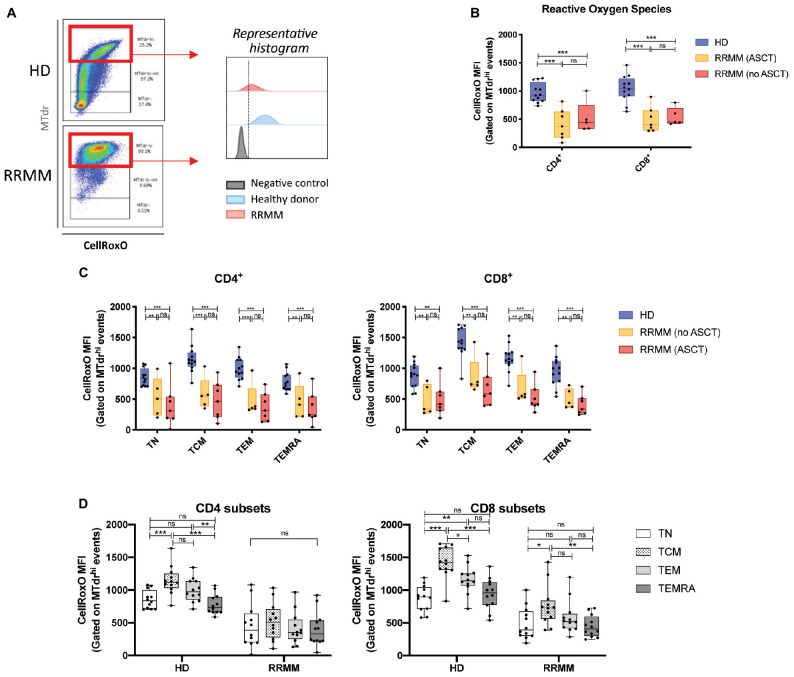
Reactive oxygen species (ROS) levels in RRMM. **(A)** Gating strategy for assessment of ROS in CD4^+^ and CD8^+^ T cells with high mitochondrial mass (MTdr hi cells), using geometric mean fluorescence intensity (MFI) of CellRox Orange (CellRoxO). **(B)** Box and whisker plots of CellRoxO MFI in CD4^+^ and CD8^+^ T cells from RRMM and HD. **(C)** Box and whisker plots of CellRoxO MFI in CD4^+^ and CD8^+^ T cell subsets from RRMM and HD. **(D)** Comparison of CellRoxO MFI in T cell differentiation subsets across RRMM (pooled) and HD. Study groups as described in Figure 1. Statistical significance in all figures was determined using multiple Student’s *t*-tests with the Holm–Sidak method applied (*p < 0.05, **p < 0.01, ***p < 0.001).

CD4^+^ and CD8^+^ T cells in RRMM had elevated mitochondrial mass, but these mitochondria may not necessarily be functional. To assess the functionality of mitochondria, we then measured ROS, which are produced during normal mitochondrial respiration, using the CellROX Orange dye. We gated on cells with high mitochondrial mass for internal consistency (Figure 6A) and saw that both CD4^+^ and CD8^+^ T cells from RRMM patients showed significantly lower ROS levels than HD (Figure 6B). This reduction in ROS levels was evident across all T cell populations (Figure 6C). In HD, ROS levels were highest in T_CM_ and T_EM_ cells, which would be expected as these cells rely more heavily on mitochondrial respiration to meet their energy demands, but in RRMM, this difference was reduced (Figure 6D).

### Mitochondrial Membrane Potential (Δψm) Is Increased in CD4^+^ T_CM_ Cells in RRMM

**FIGURE 7:**
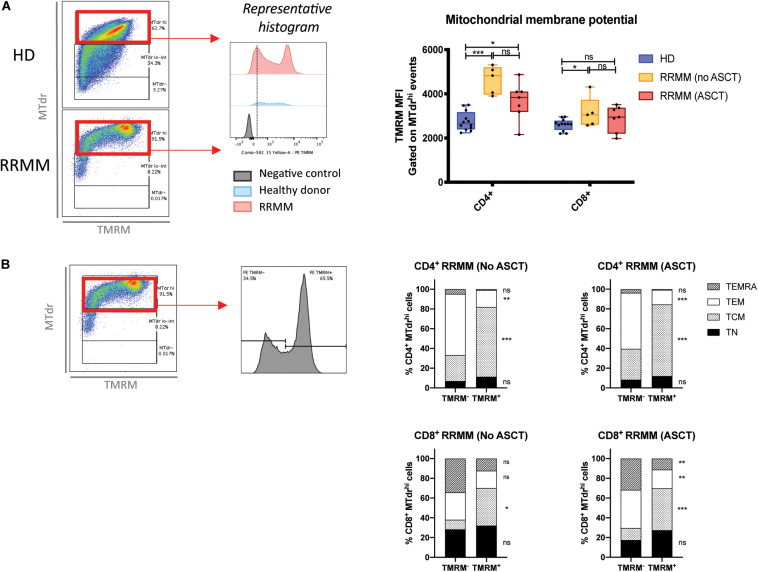
Mitochondrial membrane potential (Δψm) in T cell populations in RRMM. Mitochondrial membrane potential was measured in T cell subsets by geometric mean fluorescence intensity (MFI) of TMRM. **(A)** TMRM MFI in CD4^+^ and CD8^+^ T cells from healthy donors and RRMM: (left) Gating strategy and (right) scatter plots. **(B)** CD4^+^ and CD8^+^ T cells with high mitochondrial mass (MTdr staining) from RRMM were further subdivided into TMRM^–^ and TMRM^+^ events (gating strategy to the left) and backgated into T cell differentiation subsets, as shown in stacked bar charts of the mean frequency (right). Study groups as described in Figure 1. Statistical significance was determined using multiple Student’s *t*-tests with the Holm–Sidak method applied (*p < 0.05, **p < 0.01, ***p < 0.001).

High mitochondrial mass accompanied by low ROS levels is suggestive of reduced mitochondrial activity. To assess whether mitochondria were still functional, we assessed mitochondrial membrane potential (Δψm) using the tetramethylrhodamine, methyl ester dye (TMRM), which is retained in mitochondria with intact membrane potential. We again gated on cells with high mitochondrial mass for internal consistency and saw that Δψm was significantly higher in the CD4^+^ T cells from RRMM patients compared to HD, but there was no significant difference for CD8^+^ T cells (Figure 7A). The RRMM population appeared to be bimodal and, to compare the phenotypic composition of these two subpopulations, we back-gated TMRM^–^ and TMRM^+^ events (Figure 7B, left). A high proportion of TMRM^+^ T cells were T_CM_ phenotype, particularly within the CD4^+^ T cell population (Figure 7B, right), suggesting that T_CM_ cells are the most metabolically active subset.

## Discussion

Autologous stem cell transplant remains a backbone of MM treatment in medically fit patients, but our findings suggest that ASCT and lymphopenia-inducing therapies in MM have a largely irrecoverable effect on the CD4^+^ T cell population. We illustrate that these therapies contribute toward a reduction in the CD4:8 ratio (Figure 1) and CD4^+^ T_N_ cells (Figure 2), with a skewing of the T cell population toward a predominance of CD8^+^ T_EM_ and T_EMRA_ cells, thereby accelerating changes seen with immunosenescence. Prior analysis of these trial populations at baseline additionally identified increased IFNγ production in RRMM, largely from CD8^+^ T cells (23), which would be consistent with our observation of a predominance of CD8^+^ T_EM_ and T_EMRA_ cells. The paucity of new thymic emigrants and a heavy reliance on homeostatic proliferation of oligoclonal CD8^+^ T cells to reconstitute the T cell pool will likely significantly affect an individual’s ability to respond to new or recall antigens, whether in the form of vaccination or infection. Analysis of the molecular biomarker p16^INK4a^ (p16) suggested that ASCT induces molecular aging of T cells (37), providing further evidence of the detrimental effect of ASCT.

We acknowledge some limitations in our analysis including the restriction of T cell analysis to the PB compartment [although other studies have pointed to similarities in T cell populations from the PB and BM (23, 38)], the superiority of newer technologies such as mass spectrometry to better identify canonical populations, and the low numbers of samples in the RRMM group.

Nevertheless, our findings are particularly relevant for the utility and production of autologous CAR-T cells in MM. It appears that CAR-T cell production is best achieved with relatively undifferentiated T cells, such as T_N_ and stem cell memory subsets (39, 40). In mouse models of lymphoma, CAR-T cells derived from T_N_ and T_CM_ cells were shown to be more efficacious than those from than T_EM_ cells due to superior cytokine production of CD4^+^ T cells and cytotoxicity of CD8^+^ T cells (41). Furthermore, the potency of CAR-T cell therapy is dramatically enhanced by generating CD8^+^ CAR-T cells from T_CM_ cultured with CD4^+^ T cells. In humans, Garfall et al. (42) found that a higher frequency of CD8^+^CD45RO^–^CD27^+^ T cells and a higher CD4:8 ratio at the time of leukapheresis for CAR-T production were the key factors associated with superior clinical outcome. Therefore, a CAR-T product generated from T cells from a heavily pre-treated, immunosenescent individual would be expected to be impaired. A practical approach in the clinic to maximize the effectiveness of a CAR-T cell product in MM patients could be to collect and store T cells prior to ASCT, before the shift in composition of the T cell population, when a higher number of naïve and stem cell memory T cells might be collected.

It was, however, interesting to note that our findings of an increase in the proportion of CD4^+^ T cells with a T_CM_ phenotype in LEN/DEX-treated RRMM (Figure 2C) were also mirrored in the QUIREDEX trial (LEN ± DEX in high-risk smoldering MM) (43). This trial reported a significant increase in the proportion of CD4^+^ T_CM_ cells by cycle 9 of treatment and, moreover, CD4^+^ T cells were both functional in terms of cytokine production and proliferative as assessed by expression of activation and proliferation markers. It is not clear, however, whether this change is indicative of re-engagement of the immune system in disease control and/or is related to lenalidomide treatment, and we await the long-term outcome of this trial.

We also show that the frequency of PD1^+^CD4^+^ T cells increases post-ASCT and in RRMM, particularly within the CD4^+^ T_CM_ cell subset (Figure 3), which would be expected to impede tumor surveillance and DC vaccination. In previous work, we demonstrated that the frequency of T_REGS_ increases post-ASCT (Supplementary Figure 5) and with LEN/DEX in RRMM (44), which could upregulate PD-L1 expression on DCs. However, increased PD-1 expression can also indicate proliferation, as PD-1 is upregulated on initial T cell activation and its expression increases with sustained TCR signaling (45) or chronic antigen stimulation due to infection or tumor. To explore this possibility, further analysis of epitope specificity, TCR repertoire, and telomere length would be of interest. It is possible that a large proportion of these cells are CMV-specific, as oligoclonal expansions can develop in older humans (46), but it is also plausible that this population contains MM-specific T cell clones, the presence of which have been associated with long-term survival (47, 48).

Transcriptional and metabolic analyses of CD4^+^ and CD8^+^ T cells in RRMM showed significant increases in FAO transcripts (Figure 4) and increased mitochondrial mass (Figure 5). Memory T cells are known to have higher mitochondrial mass and engage FAO for their energy requirements, while T_N_ cells have low mitochondrial mass and low energy requirements (34). Accordingly, the shift in cell proportions after ACST, with a loss of T_N_ cells and an increase in T_CM/EM_ cells, is likely to account for some of the increase in FAO transcripts and mitochondrial mass. However, it should be noted that an increase in mitochondrial mass was observed at all T cell differentiation states. This global increase could be driven by lymphopenia-associated IL-15 signaling, as IL-15 is known to promote *CPT1a* expression and FAO engagement and promote mitochondrial biogenesis (49, 50). Mitochondrial mass was highest in T_CM_ cells and, notably, T_N_ cells. This suggests that the remaining T_N_ cells are not truly naïve and they may have acquired some memory-associated metabolic characteristics during ASCT, likely from lymphopenia-associated signals.

Given the increased mitochondrial mass in T cells in RRMM, the reduction in ROS levels was unexpected (Figure 6). A potential explanation for discordance between mitochondrial mass and ROS production is that the mitochondria that accumulate in RRMM T cells may be less metabolically fit. This is consistent with the relatively low Δψm that was observed in CD8^+^ T cells with RRMM (Figure 7A). This effect may again be driven by lymphopenia-induced IL-15 signaling, as it was recently shown that effector CD8^+^ T cells transferred into irradiated mice had increased mitochondrial content but lower Δψm compared with those transferred into IL-15-deficient irradiated mice (13). In contrast, RRMM CD4^+^ T cells exhibited elevated Δψm compared with HD but high Δψm associated with the T_CM_ subset (Figure 7B), highlighting that CD4^+^ T_CM_ remain a metabolically active subset. Clarification of the metabolic fitness of these cell subsets with further metabolic studies would be beneficial. Unfortunately, this was not feasible in this analysis due to the effects on mitochondria by lengthy cryopreservation of these samples and limited duplicate samples but would be important in future prospective studies.

## Conclusion

Taken together, our findings show that the composition and metabolic profile of the T cell compartment shifts significantly over time in MM. This is likely a consequence of an interplay between aging, duration of disease, and cumulative treatment, but ASCT clearly has a dominant role. Given the inherent variability in these processes, the use of phenotypic and metabolic biomarkers to immunologically profile a patient at the time of clinical decision making will aid in directing and optimizing therapy for MM. Furthermore, if metabolic remodeling can be attenuated without reducing reconstitution after ASCT, it may better preserve functionality in T cell populations.

## Data Availability Statement

The raw data supporting the conclusions of this article will be made available by the authors, without undue reservation. The RNAseq data is accessible under accession number PRJNA658707 at https://www.ncbi.nlm.nih.gov/bioproject/PRJNA658707/.

## Ethics Statement

The studies involving human participants were reviewed and approved by Peter MacCallum Cancer Centre. The patients/participants provided their written informed consent to participate in this study.

## Author Contributions

RC designed and performed the laboratory research, analyzed the data, and wrote the manuscript. KQ provided significant assistance with writing. SH, HQ, HP, and DR were principal investigators for the Litvacc and/or RevLite clinical trials. RK and DR assisted with the research design and editing of the manuscript. All authors contributed to the article and approved the submitted version.

## Conflict of Interest

The authors declare that the research was conducted in the absence of any commercial or financial relationships that could be construed as a potential conflict of interest.

## References

[1] Rajkumar SV Dimopoulos MA Palumbo A Bladé J Merlini G Mateos M-V International myeloma working group updated criteria for the diagnosis of multiple myeloma. *Lancet Oncol.* (2014) 15:e538–48. 10.1016/S1470-2045(14)70442-5 25439696

[2] Röllig C Knop S Bornhäuser M. Multiple myeloma. *Lancet.* (2015) 385:2197–208. 25540889 10.1016/S0140-6736(14)60493-1

[3] Turesson I Velez R Kristinsson SY Landgren O. Patterns of multiple myeloma during the past 5 decades: stable incidence rates for all age groups in the population but rapidly changing age distribution in the clinic. *Mayo Clin Proc.* (2010) 85:225–30. 10.4065/mcp.2009.0426 20194150 PMC2843108

[4] Harrison SJ Quach H Link E Seymour JF Ritchie DS Ruell S A high rate of durable responses with romidepsin, bortezomib, and dexamethasone in relapsed or refractory multiple myeloma. *Blood.* (2011) 118:6274–83. 10.1182/blood-2011-03-339879 21911830

[5] Costa LJ Brill IK Omel J Godby K Kumar SK Brown EE. Recent trends in multiple myeloma incidence and survival by age, race, and ethnicity in the United States. *Blood Adv.* (2017) 1:282–7. 10.1182/bloodadvances.2016002493 29296944 PMC5727774

[6] Herndler-Brandstetter D Ishigame H Flavell RA. How to define biomarkers of human T cell aging and immunocompetence? *Front Immunol.* (2013) 4:136. 10.3389/fimmu.2013.00136 23761794 PMC3671361

[7] Nikolich-Žugich J. Aging of the T cell compartment in mice and humans: from no naive expectations to foggy memories. *J Immunol.* (2014) 193:2622–9. 10.4049/jimmunol.1401174 25193936 PMC4157314

[8] Williams KM Hakim FT Gress RE. T cell immune reconstitution following lymphodepletion. *Semin Immunol.* (2007) 19:318–30. 10.1016/j.smim.2007.10.004 18023361 PMC2180244

[9] Douek DC McFarland RD Keiser PH Gage EA Massey JM Haynes BF Changes in thymic function with age and during the treatment of HIV infection. *Nature.* (1998) 396:690–5. 10.1038/25374 9872319

[10] Mackall CL Stein D Fleisher TA Brown MR Hakim FT Bare CV Prolonged CD4 depletion after sequential autologous peripheral blood progenitor cell infusions in children and young adults. *Blood.* (2000) 96:754–62. 10.1182/blood.v96.2.754 10887145

[11] Zhang X Sun S Hwang I Tough DF Sprent J. Potent and selective stimulation of memory-phenotype CD8+ T cells in vivo by IL-15. *Immunity.* (1998) 8:591–9. 10.1016/s1074-7613(00)80564-6 9620680

[12] Le Saout C Luckey MA Villarino AV Smith M Hasley RB Myers TG IL-7–dependent STAT1 activation limits homeostatic CD4+ T cell expansion. *JCI Insight.* (2017) 2:e96228. 10.1172/jci.insight.96228 PMC5752389 29202461

[13] Xu A Bhanumathy KK Wu J Ye Z Freywald A Leary SC IL-15 signaling promotes adoptive effector T-cell survival and memory formation in irradiation-induced lymphopenia. *Cell Biosci.* (2016) 6:30. 10.1186/s13578-016-0098-2 PMC4858849 27158441

[14] Klarquist J Chitrakar A Pennock ND Kilgore AM Blain T Zheng C Clonal expansion of vaccine-elicited T cells is independent of aerobic glycolysis. *Sci Immunol.* (2018) 3:eaas9822. 10.1126/sciimmunol.aas9822 30194241 PMC6251947

[15] Davenport B Eberlein J van der Heide V Jhun K Nguyen TT Victorino F Aging of antiviral CD8+ memory T cells fosters increased survival, metabolic adaptations, and lymphoid tissue homing. *J Immunol Am Assoc Immunol.* (2019) 202:460–75. 10.4049/jimmunol.1801277 30552164 PMC6358025

[16] Goronzy JJ Li G Yang Z Weyand CM. The janus head of T cell aging – autoimmunity and immunodeficiency. *Front Immunol.* (2013) 4:131. 10.3389/fimmu.2013.00131 23761790 PMC3671290

[17] Henson SM Lanna A Riddell NE Franzese O Macaulay R Griffiths SJ p38 signaling inhibits mTORC1-independent autophagy in senescent human CD8(+) T cells. *J Clin Invest.* (2014) 124:4004–16. 10.1172/jci75051 25083993 PMC4151208

[18] Lanna A Gomes DCO Muller-Durovic B McDonnell T Escors D Gilroy DW A sestrin-dependent Erk-Jnk-p38 MAPK activation complex inhibits immunity during aging. *Nat Immunol.* (2017) 18:354–63. 10.1038/ni.3665 28114291 PMC5321575

[19] Harmer D Falank C Reagan MR. Interleukin-6 interweaves the bone marrow microenvironment, bone loss, and multiple myeloma. *Front Endocrinol.* (2019) 9:3397. 10.3389/fendo.2018.00788 30671025 PMC6333051

[20] Joshua DE Brown RD Ho PJ Gibson J. Comprehensive review. *Clini Lymphoma Myeloma.* (2011) 8:283–6. 10.3816/CLM.2008.n.039 18854282

[21] Suen H Brown R Yang S Ho PJ Gibson J Joshua D. The failure of immune checkpoint blockade in multiple myeloma with PD-1 inhibitors in a phase 1 study. *Leukemia.* (2015) 29:1621–2. 10.1038/leu.2015.104 25987102

[22] Cooke RE Koldej R Ritchie D. Immunotherapeutics in multiple myeloma: how can translational mouse models help? *J Oncol.* (2019) 2019:18. 10.1155/2019/2186494 PMC6481018 31093282

[23] Cooke RE Gherardin NA Harrison SJ Quach H Godfrey DI Prince M Spontaneous onset and transplant models of the Vk^∗^MYC mouse show immunological sequelae comparable to human multiple myeloma. *J Transl Med.* (2016) 14:259. 10.1186/s12967-016-0994-6 PMC5011922 27599546

[24] Gherardin NA. *Unconventional T Cells: from Basic Biology to Multiple Myeloma. Minerva Access.* (2016). Available online at: http://hdl.handle.net/11343/59299

[25] Kay NE. Blood levels of immune cells predict survival in myeloma patients: results of an Eastern Cooperative Oncology Group phase 3 trial for newly diagnosed multiple myeloma patients. *Blood.* (2001) 98:23–8. 10.1182/blood.v98.1.23 11418458

[26] Harrison SJ Khot AS Tsin T Hsu AK Chen K Loudovaris M Low dose lenalidomide and dexamethasone induction followed by autologous transplantation in untreated patients with myeloma is associated with high response rates and preservation of CD8, but not CD4 or NK cellular immunity. *Blood.* (2011) 118:1862 10.1182/blood.v118.21.1862.1862

[27] Hsu AK Quach H Tai T Prince HM Harrison SJ Trapani JA The immunostimulatory effect of lenalidomide on NK-cell function is profoundly inhibited by concurrent dexamethasone therapy. *Blood.* (2011) 117:1605–13. 10.1182/blood-2010-04-278432 20978269

[28] Hsu AK Gherardin N Quach H Harrison SJ Prince HM Ritchie D. CD57+ NK CELLS ARE increased in patients with multiple myeloma and ARE primed effectors for ADCC, but NOT natural cytotoxicty. Neeson P, editor. *Blood.* (2013) 122:1904 10.1182/blood.v122.21.1904.1904

[29] Liu R Holik AZ Su S Jansz N Chen K Leong HS Why weight? Modelling sample and observational level variability improves power in RNA-seq analyses. *Nucleic Acids Res.* (2015) 43:e97. 10.1093/nar/gkv412 25925576 PMC4551905

[30] Chung DJ Pronschinske KB Shyer JA Sharma S Leung S Curran SA T-cell exhaustion in multiple myeloma relapse after autotransplant: optimal timing of immunotherapy. *Cancer Immunol Res.* (2016) 4:61–71 10.1158/2326-6066.cir-15-0055 26464015 PMC4703436

[31] Hallett WHD Jing W Drobyski WR Johnson BD. Immunosuppressive effects of multiple myeloma are overcome by PD-L1 blockade. *Biol Blood Marrow Transplant.* (2011) 17:1133–45. 10.1016/j.bbmt.2011.03.011 21536144

[32] Kearl TJ Jing W Gershan JA Johnson BD. Programmed death receptor-1/programmed death receptor ligand-1 blockade after transient lymphodepletion to treat myeloma. *J Immunol.* (2013) 190:5620–8. 10.4049/jimmunol.1202005 23616570 PMC3891840

[33] Tkachev V Goodell S Opipari AW Hao L-Y Franchi L Glick GD Programmed death-1 controls T cell survival by regulating oxidative metabolism. *J Immunol.* (2015) 194:5789–800. 10.4049/jimmunol.1402180 25972478 PMC4562423

[34] Man K Kallies A. Synchronizing transcriptional control of T cell metabolism and function. *Nat Rev Immunol.* (2015) 15:574–84. 10.1038/nri3874 26272293

[35] O’Neill LAJ Kishton RJ Rathmell J. A guide to immunometabolism for immunologists. *Nat Rev Immunol.* (2016) 16:553–65. 10.1038/nri.2016.70 27396447 PMC5001910

[36] Buck MD O’Sullivan D Pearce EL. T cell metabolism drives immunity. *J Exp Med.* (2015) 212:1345–60. 10.1084/jem.20151159 26261266 PMC4548052

[37] Rosko A Hofmeister C Benson D Efebera Y Huang Y Gillahan J Autologous hematopoietic stem cell transplant induces the molecular aging of T-cells in multiple myeloma. *Bone Marrow Transplant.* (2015) 50:1379–81. 10.1038/bmt.2015.143 26121107 PMC4821192

[38] Kourelis TV Villasboas JC Jessen E Dasari S Dispenzieri A Jevremovic D Mass cytometry dissects T cell heterogeneity in the immune tumor microenvironment of common dysproteinemias at diagnosis and after first line therapies. *Blood Cancer J.* (2019) 9:72. 10.1038/s41408-019-0234-4 PMC6713712 31462637

[39] Crompton JG Clever D Vizcardo R Rao M Restifo NP. Reprogramming antitumor immunity. *Trends Immunol.* (2014) 35:178–85. 10.1016/j.it.2014.02.003 24661777 PMC4373650

[40] Sukumar M Kishton RJ Restifo NP. Metabolic reprograming of anti-tumor immunity. *Curr Opin Immunol.* (2017) 46:14–22. 10.1016/j.coi.2017.03.011 28412583 PMC6327315

[41] Sommermeyer D Hudecek M Kosasih PL Gogishvili T Maloney DG Turtle CJ Chimeric antigen receptor-modified T cells derived from defined CD8+ and CD4+ subsets confer superior antitumor reactivity in vivo. *Leukemia.* (2015) 30:492–500. 10.1038/leu.2015.247 26369987 PMC4746098

[42] Garfall AL Dancy EK Cohen AD Hwang W-T Fraietta JA Davis MM T-cell phenotypes associated with effective CAR T-cell therapy in postinduction vs relapsed multiple myeloma. *Blood Adv.* (2019) 3:2812–5. 10.1182/bloodadvances.2019000600 31575532 PMC6784521

[43] Paiva B Mateos M-V Sanchez-Abarca LI Puig N Vidriales M-B López-Corral L Immune status of high-risk smoldering multiple myeloma patients and its therapeutic modulation under LenDex: a longitudinal analysis. *Blood.* (2016) 127:1151–62. 10.1182/blood-2015-10-662320 26668134

[44] Quach H Ritchie D Neeson P Harrison S Tai T Tainton K Regulatory T cells (Treg) are depressed in patients with relapsed/refractory multiple myeloma (MM) and increases towards normal range in responding patients treated with lenalidomide (LEN). *Blood.* (2008) 112:1696–1696. 10.1182/blood.v112.11.1696.169618544684

[45] Chikuma S Terawaki S Hayashi T Nabeshima R Yoshida T Shibayama S PD-1-mediated suppression of IL-2 production induces CD8+ T cell anergy in vivo. *J Immunol.* (2009) 182:6682–9. 10.4049/jimmunol.0900080 19454662

[46] Tu W Rao S. Mechanisms underlying T cell immunosenescence: aging and cytomegalovirus infection. *Front Microbiol.* (2016) 7:2111. 10.3389/fmicb.2016.02111 28082969 PMC5186782

[47] Brown RD Spencer A Joy Ho P Kennedy N Kabani K Yang S Prognostically significant cytotoxic T cell clones are stimulated after thalidomide therapy in patients with multiple myeloma. *Leuk Lymphoma.* (2009) 50:1860–4. 10.3109/10428190903216804 19883313

[48] Bryant C Suen H Brown R Yang S Favaloro J Aklilu E Long-term survival in multiple myeloma is associated with a distinct immunological profile, which includes proliferative cytotoxic T-cell clones and a favourable Treg/Th17 balance. *Blood Cancer J.* (2013) 3:e148. 10.1038/bcj.2013.34 24036947 PMC3789202

[49] van der Windt GJW Everts B Chang C-H Curtis JD Freitas TC Amiel E Mitochondrial respiratory capacity is a critical regulator of CD8+ T cell memory development. *Immunity.* (2012) 36:68–78. 10.1016/j.immuni.2011.12.007 22206904 PMC3269311

[50] O’Sullivan D van der Windt GJW Huang SC-C Curtis JD Chang C-H Buck MD Memory CD8(+) T cells use cell-intrinsic lipolysis to support the metabolic programming necessary for development. *Immunity.* (2014) 41:75–88. 10.1016/j.immuni.2014.06.005 25001241 PMC4120664

