# Supplementary material for: Conventional Treatment for Multiple Myeloma Drives Premature Aging Phenotypes and Metabolic Dysfunction in T Cells
Source: Front Immunol. 2020 Sep 3;11:2153. doi: 10.3389/fimmu.2020.02153 (PMC7494758; doi:10.3389/fimmu.2020.02153)
Supplement: Supplementary file 1 [file Table_1.DOCX]

Supplementary Table 1: NDMM (LITVACC trial) patient and healthy donor characteristics

**Abbreviations:** C4 cycle 4 induction, M4 post-ASCT maintenance cycle 4, EOT end of treatment, HD healthy donor, DC dendritic cell, LEN lenalidomide, DEX dexamethasone, CR complete remission, VGPR very good partial response, PR partial response, MR minor response, SD stable disease

| **Parameter** | **C4 (n=29*)** | **M4 (n=21)** | **EOT (n=21)** | **HD (n=10)** |
| --- | --- | --- | --- | --- |
| Mean age | 57 years  [43-70 yrs] | 58 years  [43-70 yrs] | 56 years  [43-69 yrs] | 65 years  [54-71 yrs] |
| Sex | Male=15, Female=13 | Male=13, Female=8 | Male=12, Female=9 | Male=4, Female=6 |
| International staging system (ISS) score | 1=18  2=8  3=2 | 1=12  2=8  3=1 | 1=13  2=8  3=0 |  |
| Post ASCT treatment arm | DC vaccine=9  LEN/DEX=19 | DC vaccine=8  LEN/DEX=13 | DC vaccine=8  LEN/DEX=13 |  |
| Response at C4 | CR=2  VGPR=5  PR=16  MR/SD=5 | CR=1  VGPR=3  PR=14  MR/SD=3 | CR=1  VGPR=3  PR=14  MR/SD=3 |  |
| Response post ASCT | CR=7  VGPR=8  PR=11  MR/SD=2 | CR=5  VGPR=8  PR=6  MR/SD=2 | CR=5  VGPR=6  PR=9  MR/SD=1 |  |
| Best response to LEN/DEX | CR=12  VGPR=9  PR=6  MR/SD=1 | CR=10  VGPR=6  PR=4  MR/SD=1 | CR=9  VGPR=8  PR=4  MR/SD=0 |  |
| Number of maintenance cycles | 15.6 [4-39] | 16.9 [4-39] | 15.8 [4-39] |  |

**Patient information missing for 1 sample*

Supplementary Table 2: RRMM (REVLITE trial) patient and healthy donor characteristics: flow cytometric analysis

**Abbreviations:** sCR stringent complete remission, VGPR very good partial response, PR partial response, PD progressive disease

| **Parameter** | **RRMM (n=12)** | **HD (n=12)** |
| --- | --- | --- |
| Mean age | 68 years [61-77 yrs] | 66 years [54-77 yrs] |
| Sex | Male=10, Female=2 | Male=7, Female=5 |
| Mean number of prior lines of therapy | 3 [1-7] |  |
| Prior transplant | No=5, Yes=7 |  |
| Best response | sCR=2, VGPR=5, PR=3, PD=2 |  |

Supplementary Table 3: RRMM (REVLITE trial) patient and healthy donor characteristics: qPCR analysis

**Abbreviations:** sCR stringent complete remission, VGPR very good partial response, PR partial response, PD progressive disease

| **Parameter** | **RRMM (n=14)** | **Healthy donors (n=10)** |
| --- | --- | --- |
| Mean age | 63 [57-77years] | 65 [54-71years] |
| Sex | Male=10, Female=4 | Male=4, Female=6 |
| Mean number of prior lines of therapy | 3 [1-7] |  |
| Prior transplant | No=5^*^ Yes=9^**^ |  |
| Best response | sCR=3, VGPR=5, PR=4, PD=2 |  |

^*^Insufficient RNA extracted from one CD8 sample for analysis. ^**^Insufficient RNA extracted from 4 CD4 samples for analysis.
