# Supplementary material for: Conventional Treatment for Multiple Myeloma Drives Premature Aging Phenotypes and Metabolic Dysfunction in T Cells
Source: Front Immunol. 2020 Sep 3;11:2153. doi: 10.3389/fimmu.2020.02153 (PMC7494758; doi:10.3389/fimmu.2020.02153)
Supplement: Supplementary file 2 [file Presentation_1.PPTX]

## Slide 1
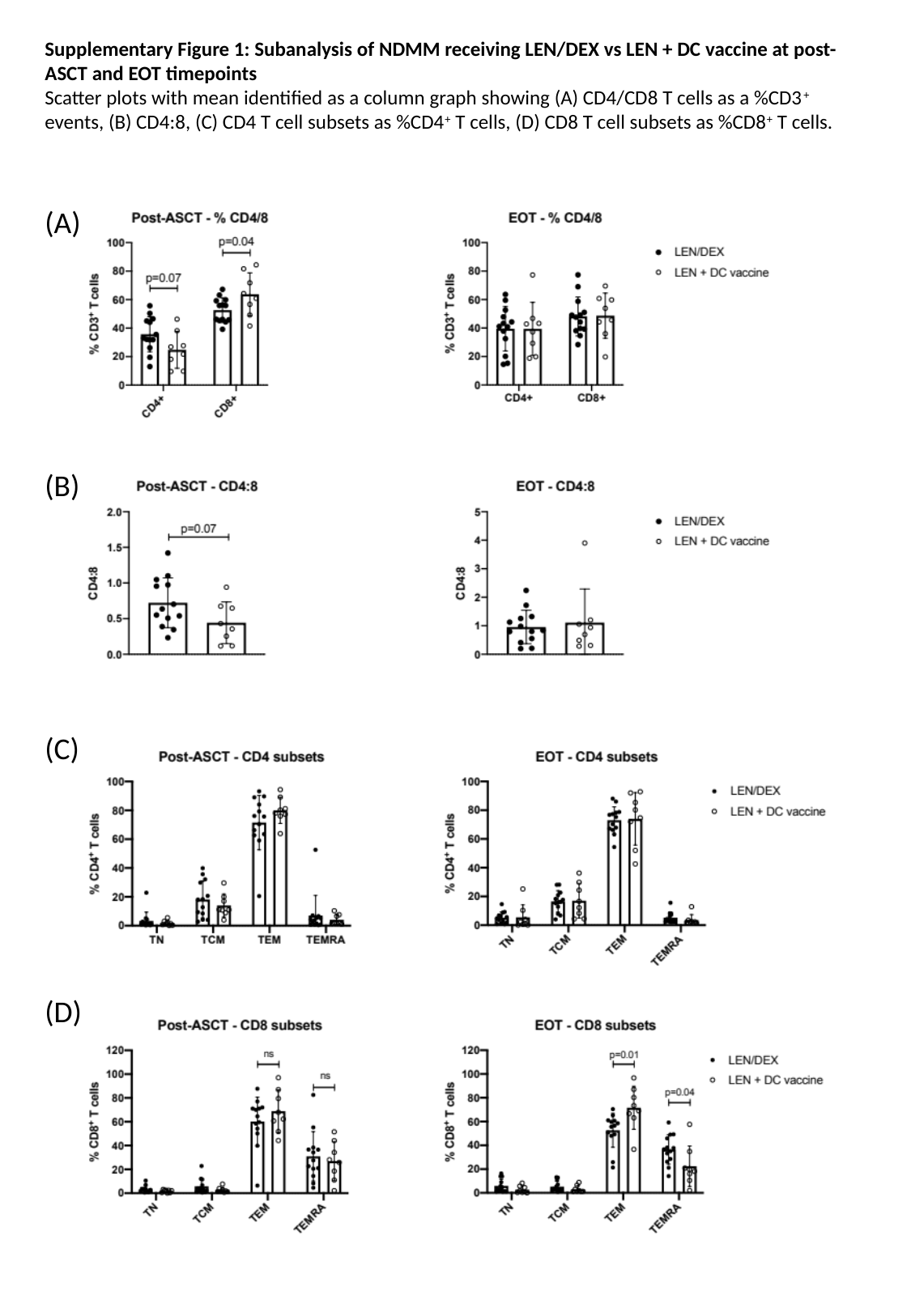

Supplementary Figure 1: Subanalysis of NDMM receiving LEN/DEX vs LEN + DC vaccine at post-ASCT and EOT timepoints
Scatter plots with mean identified as a column graph showing (A) CD4/CD8 T cells as a %CD3+ events, (B) CD4:8, (C) CD4 T cell subsets as %CD4+ T cells, (D) CD8 T cell subsets as %CD8+ T cells.
(A)
(B)
(C)
(D)
