# Supplementary material for: Conventional Treatment for Multiple Myeloma Drives Premature Aging Phenotypes and Metabolic Dysfunction in T Cells
Source: Front Immunol. 2020 Sep 3;11:2153. doi: 10.3389/fimmu.2020.02153 (PMC7494758; doi:10.3389/fimmu.2020.02153)
Supplement: Supplementary file 3 [file Presentation_2.PPTX]

## Slide 1
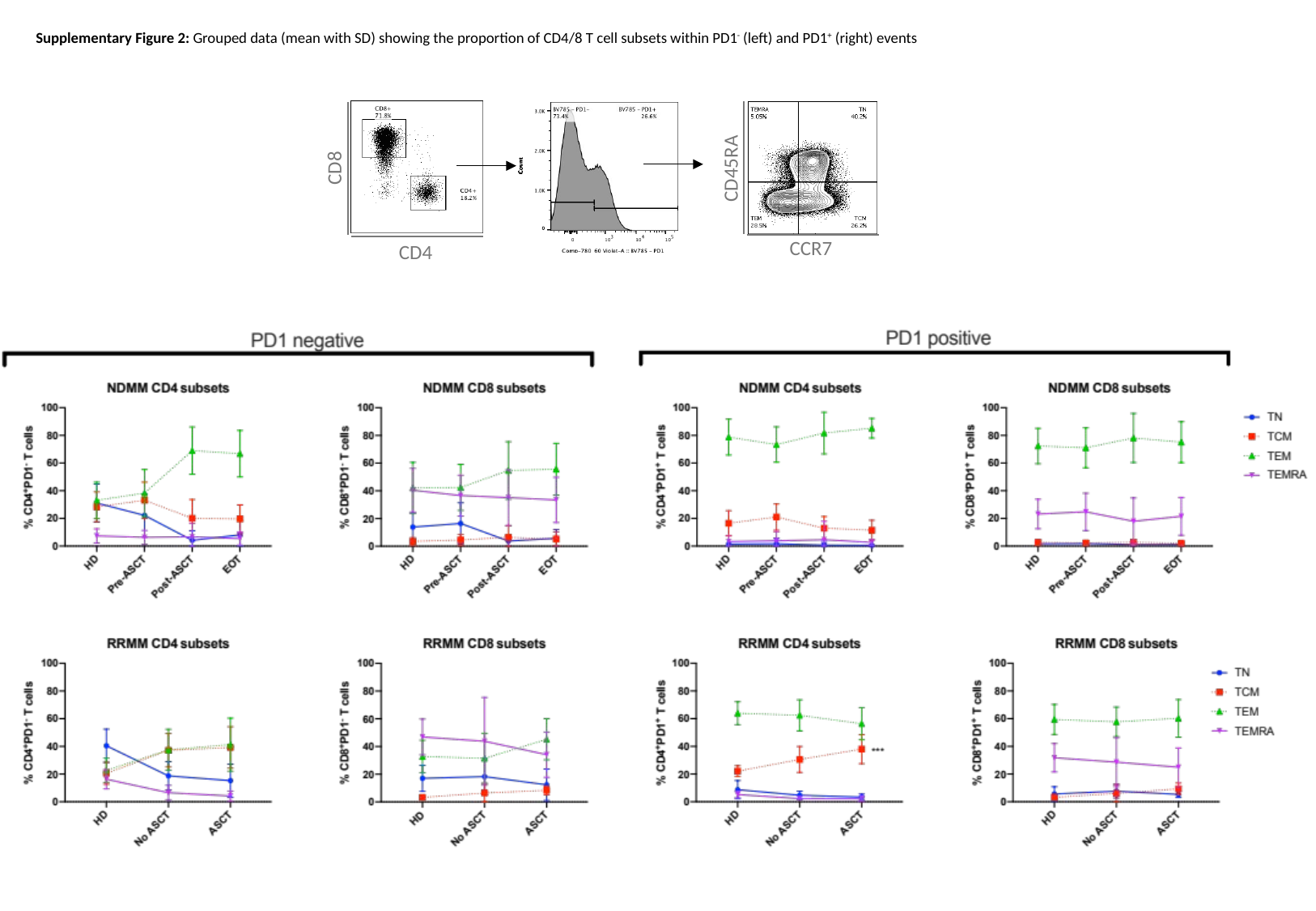

Supplementary Figure 2: Grouped data (mean with SD) showing the proportion of CD4/8 T cell subsets within PD1- (left) and PD1+ (right) events
CD45RA
CD8
CCR7
CD4
