# Supplementary material for: Conventional Treatment for Multiple Myeloma Drives Premature Aging Phenotypes and Metabolic Dysfunction in T Cells
Source: Front Immunol. 2020 Sep 3;11:2153. doi: 10.3389/fimmu.2020.02153 (PMC7494758; doi:10.3389/fimmu.2020.02153)
Supplement: Supplementary file 4 [file Presentation_3.PPTX]

## Slide 1
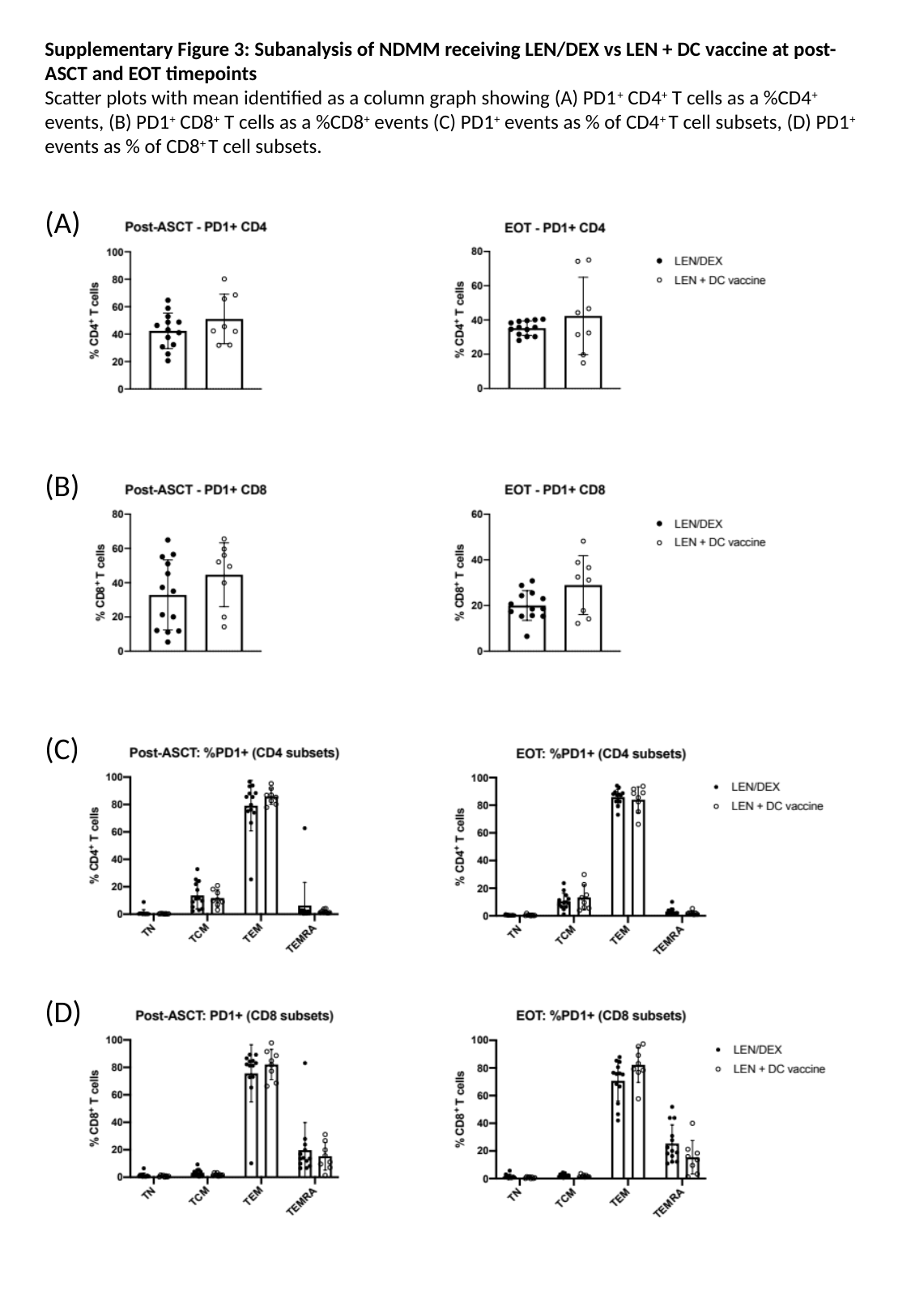

Supplementary Figure 3: Subanalysis of NDMM receiving LEN/DEX vs LEN + DC vaccine at post-ASCT and EOT timepoints
Scatter plots with mean identified as a column graph showing (A) PD1+ CD4+ T cells as a %CD4+ events, (B) PD1+ CD8+ T cells as a %CD8+ events (C) PD1+ events as % of CD4+ T cell subsets, (D) PD1+ events as % of CD8+ T cell subsets.
(A)
(B)
(C)
(D)
