# Supplementary material for: Conventional Treatment for Multiple Myeloma Drives Premature Aging Phenotypes and Metabolic Dysfunction in T Cells
Source: Front Immunol. 2020 Sep 3;11:2153. doi: 10.3389/fimmu.2020.02153 (PMC7494758; doi:10.3389/fimmu.2020.02153)
Supplement: Supplementary file 5 [file Presentation_4.PPTX]

## Slide 1
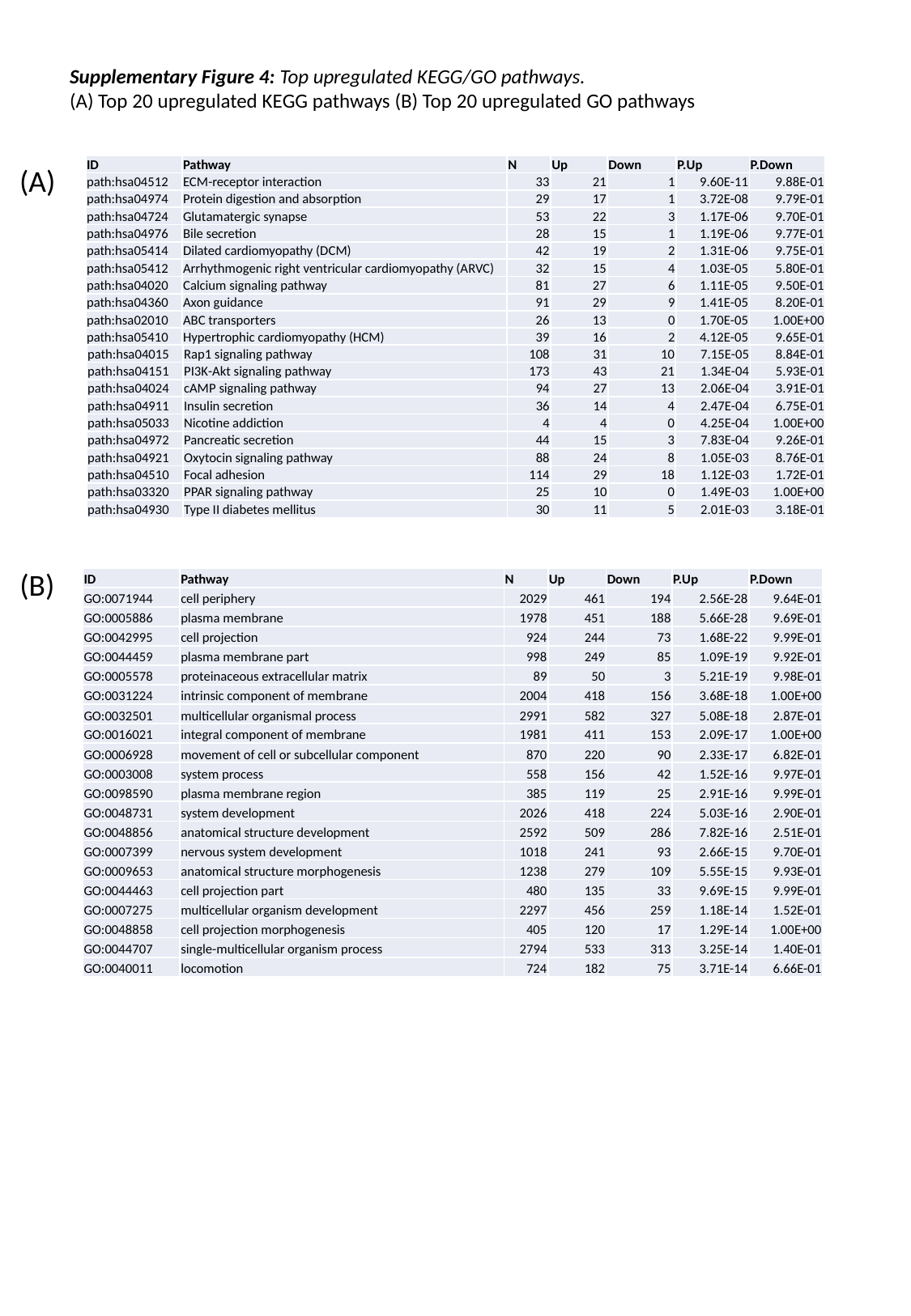

Supplementary Figure 4: Top upregulated KEGG/GO pathways.
(A) Top 20 upregulated KEGG pathways (B) Top 20 upregulated GO pathways
(A)
| ID | Pathway | N | Up | Down | P.Up | P.Down |
| --- | --- | --- | --- | --- | --- | --- |
| path:hsa04512 | ECM-receptor interaction | 33 | 21 | 1 | 9.60E-11 | 9.88E-01 |
| path:hsa04974 | Protein digestion and absorption | 29 | 17 | 1 | 3.72E-08 | 9.79E-01 |
| path:hsa04724 | Glutamatergic synapse | 53 | 22 | 3 | 1.17E-06 | 9.70E-01 |
| path:hsa04976 | Bile secretion | 28 | 15 | 1 | 1.19E-06 | 9.77E-01 |
| path:hsa05414 | Dilated cardiomyopathy (DCM) | 42 | 19 | 2 | 1.31E-06 | 9.75E-01 |
| path:hsa05412 | Arrhythmogenic right ventricular cardiomyopathy (ARVC) | 32 | 15 | 4 | 1.03E-05 | 5.80E-01 |
| path:hsa04020 | Calcium signaling pathway | 81 | 27 | 6 | 1.11E-05 | 9.50E-01 |
| path:hsa04360 | Axon guidance | 91 | 29 | 9 | 1.41E-05 | 8.20E-01 |
| path:hsa02010 | ABC transporters | 26 | 13 | 0 | 1.70E-05 | 1.00E+00 |
| path:hsa05410 | Hypertrophic cardiomyopathy (HCM) | 39 | 16 | 2 | 4.12E-05 | 9.65E-01 |
| path:hsa04015 | Rap1 signaling pathway | 108 | 31 | 10 | 7.15E-05 | 8.84E-01 |
| --- | --- | --- | --- | --- | --- | --- |
| path:hsa04151 | PI3K-Akt signaling pathway | 173 | 43 | 21 | 1.34E-04 | 5.93E-01 |
| path:hsa04024 | cAMP signaling pathway | 94 | 27 | 13 | 2.06E-04 | 3.91E-01 |
| path:hsa04911 | Insulin secretion | 36 | 14 | 4 | 2.47E-04 | 6.75E-01 |
| path:hsa05033 | Nicotine addiction | 4 | 4 | 0 | 4.25E-04 | 1.00E+00 |
| path:hsa04972 | Pancreatic secretion | 44 | 15 | 3 | 7.83E-04 | 9.26E-01 |
| path:hsa04921 | Oxytocin signaling pathway | 88 | 24 | 8 | 1.05E-03 | 8.76E-01 |
| path:hsa04510 | Focal adhesion | 114 | 29 | 18 | 1.12E-03 | 1.72E-01 |
| path:hsa03320 | PPAR signaling pathway | 25 | 10 | 0 | 1.49E-03 | 1.00E+00 |
| path:hsa04930 | Type II diabetes mellitus | 30 | 11 | 5 | 2.01E-03 | 3.18E-01 |
(B)
| ID | Pathway | N | Up | Down | P.Up | P.Down |
| --- | --- | --- | --- | --- | --- | --- |
| GO:0071944 | cell periphery | 2029 | 461 | 194 | 2.56E-28 | 9.64E-01 |
| GO:0005886 | plasma membrane | 1978 | 451 | 188 | 5.66E-28 | 9.69E-01 |
| GO:0042995 | cell projection | 924 | 244 | 73 | 1.68E-22 | 9.99E-01 |
| GO:0044459 | plasma membrane part | 998 | 249 | 85 | 1.09E-19 | 9.92E-01 |
| GO:0005578 | proteinaceous extracellular matrix | 89 | 50 | 3 | 5.21E-19 | 9.98E-01 |
| GO:0031224 | intrinsic component of membrane | 2004 | 418 | 156 | 3.68E-18 | 1.00E+00 |
| GO:0032501 | multicellular organismal process | 2991 | 582 | 327 | 5.08E-18 | 2.87E-01 |
| GO:0016021 | integral component of membrane | 1981 | 411 | 153 | 2.09E-17 | 1.00E+00 |
| GO:0006928 | movement of cell or subcellular component | 870 | 220 | 90 | 2.33E-17 | 6.82E-01 |
| GO:0003008 | system process | 558 | 156 | 42 | 1.52E-16 | 9.97E-01 |
| GO:0098590 | plasma membrane region | 385 | 119 | 25 | 2.91E-16 | 9.99E-01 |
| GO:0048731 | system development | 2026 | 418 | 224 | 5.03E-16 | 2.90E-01 |
| GO:0048856 | anatomical structure development | 2592 | 509 | 286 | 7.82E-16 | 2.51E-01 |
| GO:0007399 | nervous system development | 1018 | 241 | 93 | 2.66E-15 | 9.70E-01 |
| GO:0009653 | anatomical structure morphogenesis | 1238 | 279 | 109 | 5.55E-15 | 9.93E-01 |
| GO:0044463 | cell projection part | 480 | 135 | 33 | 9.69E-15 | 9.99E-01 |
| GO:0007275 | multicellular organism development | 2297 | 456 | 259 | 1.18E-14 | 1.52E-01 |
| GO:0048858 | cell projection morphogenesis | 405 | 120 | 17 | 1.29E-14 | 1.00E+00 |
| GO:0044707 | single-multicellular organism process | 2794 | 533 | 313 | 3.25E-14 | 1.40E-01 |
| GO:0040011 | locomotion | 724 | 182 | 75 | 3.71E-14 | 6.66E-01 |
