# Supplementary material for: Conventional Treatment for Multiple Myeloma Drives Premature Aging Phenotypes and Metabolic Dysfunction in T Cells
Source: Front Immunol. 2020 Sep 3;11:2153. doi: 10.3389/fimmu.2020.02153 (PMC7494758; doi:10.3389/fimmu.2020.02153)
Supplement: Supplementary file 6 [file Presentation_5.PPTX]

## Slide 1
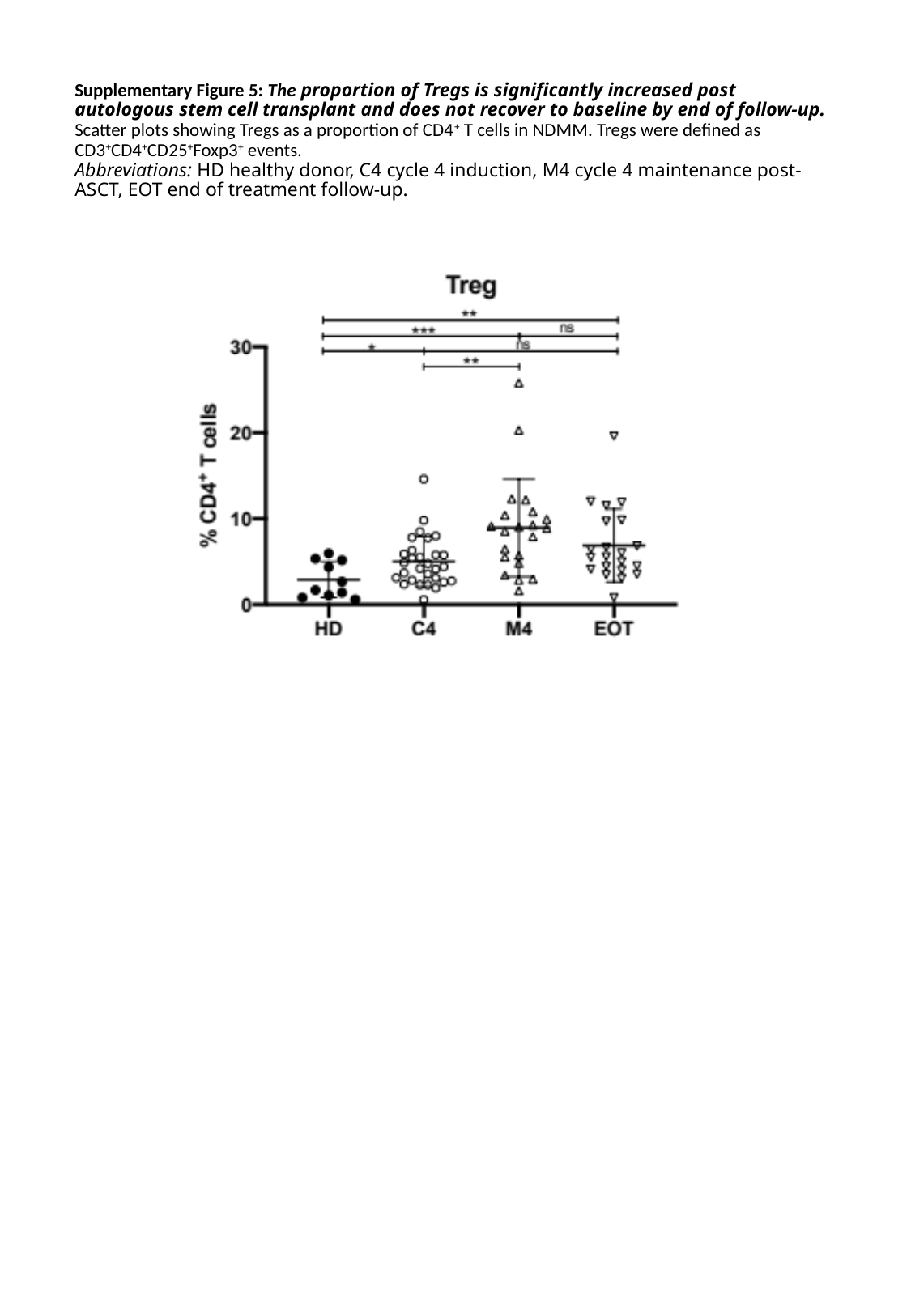

# Supplementary Figure 5: The proportion of Tregs is significantly increased post autologous stem cell transplant and does not recover to baseline by end of follow-up. Scatter plots showing Tregs as a proportion of CD4+ T cells in NDMM. Tregs were defined as CD3+CD4+CD25+Foxp3+ events. Abbreviations: HD healthy donor, C4 cycle 4 induction, M4 cycle 4 maintenance post-ASCT, EOT end of treatment follow-up.
